# A Novel GH7 Endo-β-1,4-Glucanase from *Neosartorya fischeri* P1 with Good Thermostability, Broad Substrate Specificity and Potential Application in the Brewing Industry

**DOI:** 10.1371/journal.pone.0137485

**Published:** 2015-09-11

**Authors:** Yun Liu, Baoqing Dun, Pengjun Shi, Rui Ma, Huiying Luo, Yingguo Bai, Xiangming Xie, Bin Yao

**Affiliations:** 1 College of Biological Sciences and Biotechnology, Beijing Forestry University, Beijing 100083, P. R. China; 2 Key Laboratory for Feed Biotechnology of the Ministry of Agriculture, Feed Research Institute, Chinese Academy of Agricultural Sciences, Beijing 100081, P. R. China; 3 The National Key Facility for Crop Gene Resources and Genetic Improvement, Institute of Crop Sciences, Chinese Academy of Agricultural Sciences, Beijing 100081, China; University of Melbourne, AUSTRALIA

## Abstract

An endo-β-1,4-glucanase gene, *cel7A*, was cloned from the thermophilic cellulase-producing fungus *Neosartorya fischeri* P1 and expressed in *Pichia pastoris*. The 1,410-bp full-length gene encodes a polypeptide of 469 amino acids consisting of a putative signal peptide at residues 1–20, a catalytic domain of glycoside hydrolase family 7 (GH7), a short Thr/Ser-rich linker and a family 1 carbohydrate-binding module (CBM 1). The purified recombinant Cel7A had pH and temperature optima of pH 5.0 and 60°C, respectively, and showed broad pH adaptability (pH 3.0–6.0) and excellent stability at pH3.0–8.0 and 60°C. Belonging to the group of nonspecific endoglucanases, Cel7A exhibited the highest activity on barley β-glucan (2020 ± 9 U mg^–1^), moderate on lichenan and CMC-Na, and weak on laminarin, locust bean galactomannan, Avicel, and filter paper. Under simulated mashing conditions, addition of Cel7A (99 μg) reduced the mash viscosity by 9.1% and filtration time by 24.6%. These favorable enzymatic properties make Cel7A as a good candidate for applications in the brewing industry.

## Introduction

Plant biomass consists of three major fractions: cellulose, hemicellulose and lignin [1]. As the most abundant constituent of plant biomass, cellulose is a high-molecular-weight linear polysaccharide of d-glucose linked by β-1,4 bonds that are stabilized by intermolecular and intramolecular hydrogen bonds [2]. Because of its relative insolubility and rigid structure, the complete degradation of cellulose depends on the synergistic action of several cellulolytic enzymes [3]. Cellulases are produced by many organisms, including microorganisms, plants, and animals [4, 5, 6]. Based on their amino acid sequences and substrate specificities, cellulases are divided into three major groups: endoglucanase (EC3.2.1.4), cellobiohydrolase (EC 3.2.1.91 and EC 3.2.1.176), and β-glucosidase (EC3.2.1.21) [7, 8]. Endo-β-1,4-glucanases catalyze the hydrolysis of β-1,4-glucosidic linkages in cellulose, and are grouped into 16 families of glycosyl hydrolase (GH) in Carbohydrate-Active Enzymes (CAZy) database (http://www.cazy.org/). Those from fungi are confined into 8 GH families, including GH5–7, 9, 12, 45, 48 and 74 [9].

GH7 endoglucanases have nonspecific substrate preference with the activity towards cellulose, barley β-glucan, lichenin, laminarin and xylan. This broad substrate specificity makes GH7 endoglucanases attractive for potential use in various industrial applications [10]. During malt production, the remaining high molecular weight β-glucans may cause several problems, such as increasing viscosity which impairs pumping and filtration, causing lower efficiency, and reducing yields of extracts [11]. Addition of β-glucanases to the mash could allow partial hydrolysis of barley β-glucans, and reduce both the viscosity and the filtration time, and improve yields of extracts [12]. In recent years, thermostable endo-β-glucanases have become more favorable in high temperature industries by reducing contamination risk, increasing substrate solubility, and shortening processing period [10]. Thus, exploration of new thermostable endo-β-glucanses with good properties is highly desirable.

The methylotrophic yeast *Pichia pastoris* has been developed as a high-level expression system for overproduction of heterologous proteins. This expression system has many advantages, such as employment of a simple and inexpensive medium, high production yield, simple protein processing procedure, and post-translational modification [13]. The thermophilic *Neosartorya fischeri* strain P1 was isolated from the acid wastewater of a tin mine and has the capacity to secrete a variety of GHs having some properties in common: high temperature optima, acidic pH optima, and excellent thermostability and acid stability [14, 15]. In this article, we describe the cloning of a new family 7 endo-β-1,4-glucanase gene from *N*. *fischeri* P1. The gene was successfully expressed in *P*. *pastoris*, and the purified recombinant enzyme exhibited wide substrate specificity, broad acid optimum, and good thermal and pH stability.

## Materials and Methods

### Strains, plasmids, culture conditions, and chemicals

*N*. *fischeri* P1 (CGMCC 3.15369, the China General Microbiological Culture Collection, Beijing, China) was grown in cellulase inducing medium (w/v) containing 1% wheat bran, 0.5% (NH_4_)_2_SO_4_, 0.1% KH_2_PO_4_, 0.05% MgSO_4_ 7H_2_O, 0.02% CaCl_2_ and 0.001% FeSO_4_ 7H_2_O at 45°C for 3 days. *Escherichia coli* strain Trans1-T1 (TransGen, Beijing, China) grown at 37°C in Luria-Bertani medium supplemented with ampicillin (100 μg ml^−1^) and pEASY-T3 (TransGen) were used for cloning experiment. *P*. *pastoris* strain GS115 and pPIC9 (Invitrogen, Carlsbad, CA, USA) were used for heterologous protein production. *P*. *pastoris* cells were propagated in YPD medium (yeast extract peptone dextrose medium: 1% yeast extract, 2% peptone, and 2% glucose) and subsequently grown in either MD (minimal dextrose medium: 1.34% YNB [yeast nitrogen base without amino acids], 4 × 10^−5^% biotin, 2% glucose, and 2% agarose) or BMGY (buffered glycerol-complex medium: 1% yeast extract, 2% peptone, 1.34% YNB, 4 × 10^−5^% biotin, and 1% glycerol). The induction medium was BMMY (buffered methanol-complex medium: 1% yeast extract, 2% peptone, 1.34% YNB, 4 × 10^−5^% biotin, and 0.5% methanol). Growth conditions were as described in the *Pichia* Expression kit (Invitrogen).

### Cloning and sequence analysis of *cel7A*

Mycelia of strain P1 were collected after 3 days’ growth in cellulase inducing medium as described above and then were frozen in liquid nitrogen. After grinding them into a fine powder, total RNA was extracted and purified by using the SV Total RNA Isolation System (Promega, Madison, WI, USA) according to the manufacturer’s instructions. Full-length cDNA was obtained by reverse transcription (RT)-PCR using the TransScript One-Step gDNA Removal and cDNA Synthesis SuperMix (TransGen) and two specific primers (cel-f: 5′- GGGGAATTCCAACAACCCGCCACAAGTTCTGCTGG-3′, and cel-r: 5′- GGGGCGGCCGCCTACAGACACTGTGAGTACCACTGATT-3′, restriction sites underlined) designed based on the known genome sequence of *N*. *fischeri* P1 (http://genome.jgi-psf.org/Neofi1/Neofi1.home.html). The purified PCR product was cloned into pEASY-T3 vector, transformed into *E*. *coli* DH5α cells and sequenced by Ruibo (Beijing, China). The sequences were analyzed by using the Vector NTI Suite 10.0 software (http://www.hsls.pitt.edu/guides/genetics/vectornti/) and BLASTx and BLASTp available on the NCBI web pages. The signal peptide sequence of Cel7A was predicted using SignalP 4.0 server (http://www.cbs.dtu.dk/services/SignalP/).

### Construction of the recombinant expression vector

The gene fragment coding for mature protein without the putative signal peptide was amplified by PCR with primers EGL-PF (forward, 5′- GGGGAATTCCAACAACCCGCCACAAGTTCTGCTGG-3′) and EGL-PR (reverse, 5′-GGGGCGGCCGCCTACAGACACTGTGAGTACCACTGATT-3′) containing *Eco*RI and *Not*I restriction sites (underlined), respectively. The PCR products were digested with *Eco*RI and *Not*I and ligated into *Eco*RI-*Not*I digested pPIC9 vector to construct the recombinant plasmid pPIC9-*cel7A*, in which *cel7A* was fused with the α-factor signal sequence and was under the control of AOX1 promoter. The plasmid was linearized by *Bgl*II digestion and transformed into *P*. *pastoris* GS115 competent cells by electroporation as described previously [16].

### Expression and purification of recombinant Cel7A in *P*. *pastoris*

*P*. *pastoris* GS115 cells harboring pPIC9-*cel7A* were screened on MD plates by 2 days’ growth at 30°C. Positive transformants were each grown in 3 ml of BMGY with constant agitation of 220 rpm for ~48 h. Cells were then harvested and resuspended in 1 ml of BMMY for another 48 h growth. Endoglucanase activities in the culture supernatants were determined as described below. The transformant exhibiting the highest endoglucanase activity was selected for large-scale expression as follows. The recombinant yeast cells were first grown in 50 ml of YPD (250-ml flasks) at 30°C, 220 rpm for 2 days, and 1% of the culture (approximately 4 ml) was inoculated into 400 ml of BMGY (1-l flasks) for another 2 days at 30°C and 220 rpm. Cells were harvested by centrifugation and grown in 200 ml of BMMY (1-liter flasks) as described above. Methanol was added to the culture every 24 h to a final concentration of 1% for endoglucanase induction.

The induced cultures were centrifuged (12,000 × g, 4°C) for 30 min to remove yeast cells and undissolved materials followed by ultrafiltration with Vivaflow 200 membrane of 10-kDa molecular weight cutoff (Vivascience, Hannover, Germany). The supernatants were concentrated by ultrafiltration with a Biomax M_r_ 30,000-cutoff membrane (Millipore, Billercia, MA, USA). The crude enzyme was loaded onto the HiTrap Desalting column (GE Healthcare, USA) with 20 mM McIlvaine buffer (40 mM Na_2_HPO_4_, 20 mM citric acid, pH 7.0) and HiTrap Q Sepharose XL FPLC column (GE Healthcare, USA) sequentially. Proteins were eluted by a linear gradient of NaCl (0−1.0 M) in the same buffer as described above at a flow rate of 3 ml min^–1^. The purity and molecular mass of Cel7A was checked by sodium dodecyl sulfate (SDS)-polyacrylamide gel electrophoresis (PAGE) [17]. Protein concentration was determined by the Bio-Rad protein assay kit (Waltham, MA, USA) using bovine serum albumin as the standard. To remove N-glycosylation, the purified recombinant Cel7A was treated with Endo H (New England Biolabs, Ipswitch, MA, USA) for 2 h at 37°C according to the manufacturer’s instructions. Deglycosylated Cel7A was also analyzed by SDS-PAGE.

### Endoglucanase activity assay

The endo-β-1,4-glucanase activity was determined by measuring the release of reducing sugar from barley β-glucan (Sigma-Aldrich, St. Louis, MO, USA) with the 3,5-dinitrosalicylic acid (DNS) method [18]. The standard reaction mixture, containing 100 μl of appropriately diluted enzyme and 1.0% (w/v) barley β-glucan in 900 μl of McIlvaine buffer (200 mM Na_2_HPO_4_, 100 mM citric acid, pH 5.0), was incubated in water-bath at 60°C for 10 min and terminated by the addition of 1.5 ml of DNS. After 5-min boiling, the reaction mixture was cooled down to room temperature in ice water. The absorbance was measured at 540 nm. One unit of endo-β-1,4-glucanase activity was defined as the amount of enzyme required to release 1 μmol of glucose per minute.

### Biochemical characterization of recombinant Cel7A

The enzyme activity was measured with barley β-glucan as the substrate under the specified assay conditions. The optimum pH for the activity of recombinant Cel7A was determined at 60°C for 10 min in the following buffers (100 mM): glycine-HCl (pH 2.0–3.0), NaH_2_PO_4_-citric acid (pH 3.0–8.0), Tris-HCl (pH 8.0–9.0), and glycine-NaOH (pH 9.0–12.0). To determine the pH stability, the enzyme was pre-incubated in water-bath at 37°C for 60 min in different buffers of 2.0 to 12.0 as described above, and the residual activity was measured under standard assay conditions (pH 5.0, 60°C and 10 min). The initial enzyme activity was defined as 100%.

The optimal temperature was determined in 100 mM NaH_2_PO_4_-citric acid (pH 5.0) at different temperatures ranging from 30°C to 90°C for 10 min. The thermal stability was determined by pre-incubating the enzyme at 60 or 70°C for different durations and measuring the residual activities under standard assay conditions.

The effect of metal cations and chemical reagents on the activity of recombinant Cel7A was assessed by incubating the enzyme in 100 mM NaH_2_PO_4_-citric acid (pH 5.0) containing 5 mM of NaCl, KCl, CaCl_2_, LiCl, CoCl_2_, CrCl_3_, NiCl_2_, CuCl_2_, MgCl_2_, FeCl_3_, MnCl_2_, ZnCl_2_, Pb(CH_3_COO)_2_, AgNO_3_, HgCl_2_, SDS, ethylenediaminetetraacetic acid (EDTA) or β-mercaptoethanol at room temperature for 30 min. The residual activities were determined under standard assay conditions (pH 5.0, 60°C and 10 min). The enzyme activity without any addition was set as 100%.

### Substrate specificity and kinetic parameters

Besides barley β-glucan, other substrates including sodium carboxyl methyl cellulose (CMC-Na), 4-nitrophenyl α-d-galactopyranoside (*p*NPG), 4-nitrophenyl β-cellobioside (*p*NPC), birchwood xylan, Avicel, phosphoric acid swollen cellulose (PASC) and locust bean galactomannan were all purchased from Sigma-Aldrich. Lichenin was purchased from Megazyme (Wicklow, Ireland). Whatman Grade 1 filter paper was purchased from General Electric Company, United Kingdom. The enzyme activities of Cel7A towards various substrates (1.0%, w/v) were assessed by using the standard methods. Except for filter paper that required a longer incubation period (30 min), other substrates were incubated with Cel7A for 10 min. All enzyme activity measurements were carried out three times.

The kinetic parameters *K*_*m*_ and *V*_*max*_ were determined in 100 mM McIlvaine buffer (pH 5.0) containing 0.5−10.0 mg ml^−1^ barley β-glucan at 60°C for 5 min. The *K*_*m*_ and *V*_*max*_ values were calculated according to the Lineweaver-Burk method.

### Analysis of the hydrolysis products

The hydrolysis products of barley β-glucan or CMC-Na by Cel7A catalysis were analyzed by the high-performance anion exchange chromatography (HPAEC; Thermo Fisher Scientific, Sunnyvale, CA, USA) equipped with a Carbo-Pac PA200 column (3 μm × 250 mm). Purified recombinant 100 ul Cel7A (500 U and 100 U, respectively) was incubated with 900 ul barley β-glucan (3.0 mg) or CMC-Na (1.5 mg) in McIlvaine buffer (pH 5.0) at 50°C for 12 h. Equal volume of McIlvaine buffer was added as described above as control. All reactions were terminated by 10-min boiling. The reducing sugar released was then separated and concentrated by ultrafiltration with a Biomax M_r_ 30,000-cutoff membrane (Millipore). Samples (100 ul) were properly diluted 1000 folds in ddH_2_O and 600 ul loaded on HPAEC equipped with a Carbo-Pac PA200 column. To elute the oligosaccharides, NaOH (100 mM) was used at the flow rate of 0.3 ml min^−1^. Cellooligosaccharide (glucose, cellobiose, cellotriose, cellotetraose, cellopentaose, and cellohexaose) from Seikagaku (Tokyo, Japan) were used as the standards.

### Effects of Cel7A on the reduction of mash viscosity

Mash was prepared as described by Celestino et al. with some modifications [19]. Dry malt (5 g) was triturated in a disintegrator, followed by filtration through a 0.2-mm sieve. The fine powders were dissolved in 50 ml of McIlvaine buffer (pH 5.5) as the mash. Reactions containing 50 ml of mash and 200 U of Cel7A (99 μg) or equal volume of McIlvaine buffer (the control) were incubated at 45°C for 30 min, 50°C for 30 min, 60°C for 60 min, 70°C for 15 min, and then boiled for 5 min. After centrifugation (12000 *g*, 15 min and 4°C) and filtration through filter paper, the viscosity of mash supernatant (10 ml) was measured at room temperature by a capillary viscometer. Mash viscosity in the absence of enzyme was used as a control. The viscosity reduction was calculated using the following equations [19].

μ=(μwater×t×ρ)/(twater×ρwater) (1)

Δμ=(μcontrol−μ)×100/(μcontrol) (2)

Where μ is the viscosity, t is the total flow time through viscometer, Δμ is the reduction of viscosity, and ρ is the density.

### Nucleotide sequence accession number

The nucleotide sequence of the gene *cel7A* from *N*. *fischeri* P1 was deposited in the GenBank database under accession number KP861909.

## Results

### Cloning and sequence analysis of *cel7A*

The cDNA of *cel7A* was cloned from *N*. *fischeri* P1 with specific primers. The open reading frame consists of 1,410 bp that encodes a protein of 469 amino acid residues and a termination codon. The theoretical molecular mass and isoelectric point were predicted to be 47.2 kDa and 4.87, respectively. Sequence analysis with SignalP indicated the presence of a putative 20-residue signal sequence (MDSKRGIVAAVLALLSVVSA). The mature protein contains three possible N-glycosylation sites, namely Asn 96 (Asn-Tyr-Thr), Asn 201 (Asn-Gly-Thr), and Asn 205 (Asn-Thr-Ser). BLASTx analysis revealed that deduced Cel7A has the highest identity of 99% with the hypothetical endoglucanase from *N*. *fischeri* NRRL 181 (XP_001257357.1) and of 78% with the functionally characterized endoglucanase Cel7B from *Penicillium decumbens* 114–2. Multiple sequence alignments (S1 Fig) indicated that deduced Cel7A contains the conserved active-site residues, EMDILE, of GH 7 members [20].

### Heterologous expression and purification of Cel7A

**Fig 1:**
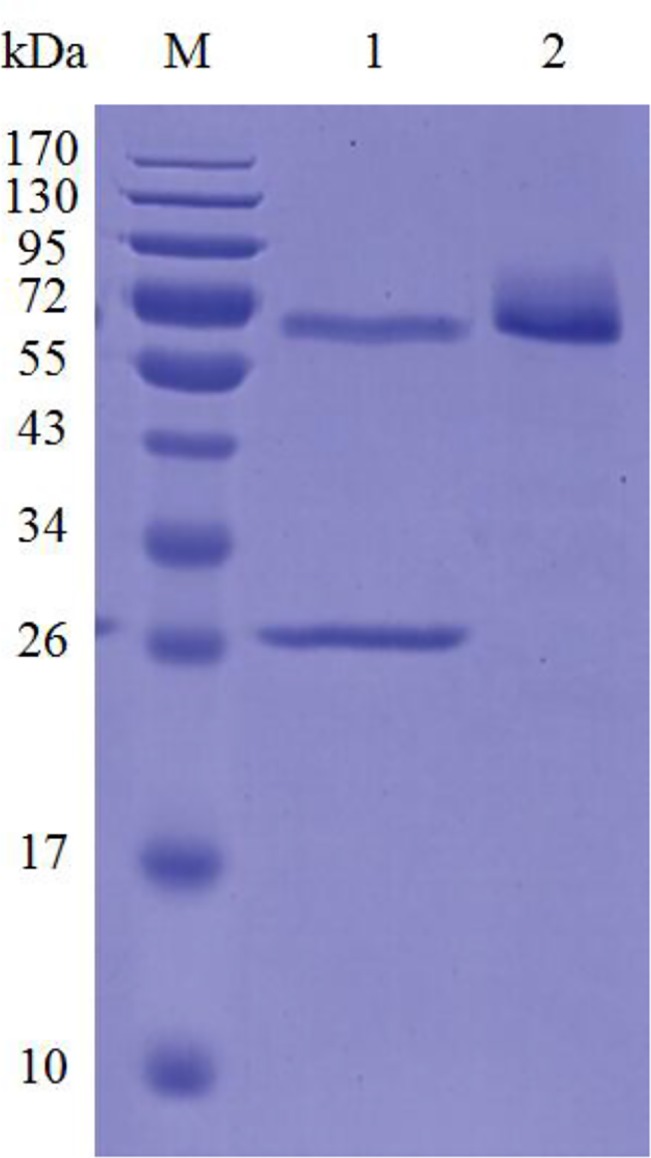
SDS-PAGE analysis of purified recombinant Cel7A. Lanes: M, the molecular mass standards; 1, the deglycosylated Cel7A with Endo H treatment; 2, the purified recombinant Cel7A.

The recombinant Cel7A was expressed in *P*. *pastoris*. When using barley β-glucan as the substrate, the enzyme activity of 1000 U ml^–1^ and protein concentration of 0.5 g l^–1^ were detected in the culture supernatants after 72-h incubation in BMMY. The recombinant enzyme in the culture supernatant was purified to electrophoretic homogeneity by one-step anion exchange chromatography (S1 Table). The apparent molecular mass of purified recombinant Cel7A was ~72 kDa, much higher than its calculated value (47.2 kDa) (Fig 1). After treatment with Endo H specific for N-glycosylation removal, the molecular mass had a slight decrease, but was still higher than its calculated value. The extra molecular masses might be ascribed to other post-translational modifications like *O*-glycosylation and hyper-glycosylation occurring during heterologous expression in yeast.

### Substrate specificity and kinetic parameters of the recombinant Cel7A

The specificity of recombinant Cel7A for glycosidic bonds was assayed against several different substrates (Table 1). Cel7A exhibited the highest activity toward barley β-glucan (2020 ± 9 U mg^–1^) followed by lichenin and CMC-Na, all of which contain β-1,4-glucan. Cel7A could also hydrolyze laminarin, locust bean galactomannan, Avicel, PASC and filter paper, although the efficiencies were much lower than with CMC-Na. No activity against *p*NPC and *p*NPG was detected.

**Table 1:** Substrate specificity of the recombinant Cel7A.

Substrate	Main linkage (monomer)	Specific activity (U mg^–1^)	Relative activity (%)
Barley β-glucan	1,3–1,4-β-(glucose)	2020 ± 9	100.0
Lichenan	1,3–1,4-β-(glucose)	1075 ± 5	53.2
CMC-Na	1,4-β-(glucose)	375 ± 3	18.6
Laminarin	1,3-β-(glucose)	185 ± 2	9.1
Locust bean galactomannan	1,4-β-(mannoose)	94 ± 2	4.7
Avicel	1,4-β-(glucose)	81 ± 1	4.0
PASC	1,4-β-(glucose)	122 ± 3	6.0
Filter paper	1,4-β-(glucose)	42 ± 1	2.1
Beechwood xylan	1,4-β-d-(xylan)	7 ± 1	0.3

The kinetic parameters were determined using barley β-glucan as the substrate. The *K*_m_ and *V*_max_ values were 4.5 ± 0.2 mg ml^–1^ and 5000 ± 186 μmol min^–1^ mg^–1^ for Cel7A.

### Biochemical characterization of recombinant Cel7A

**Fig 2:**
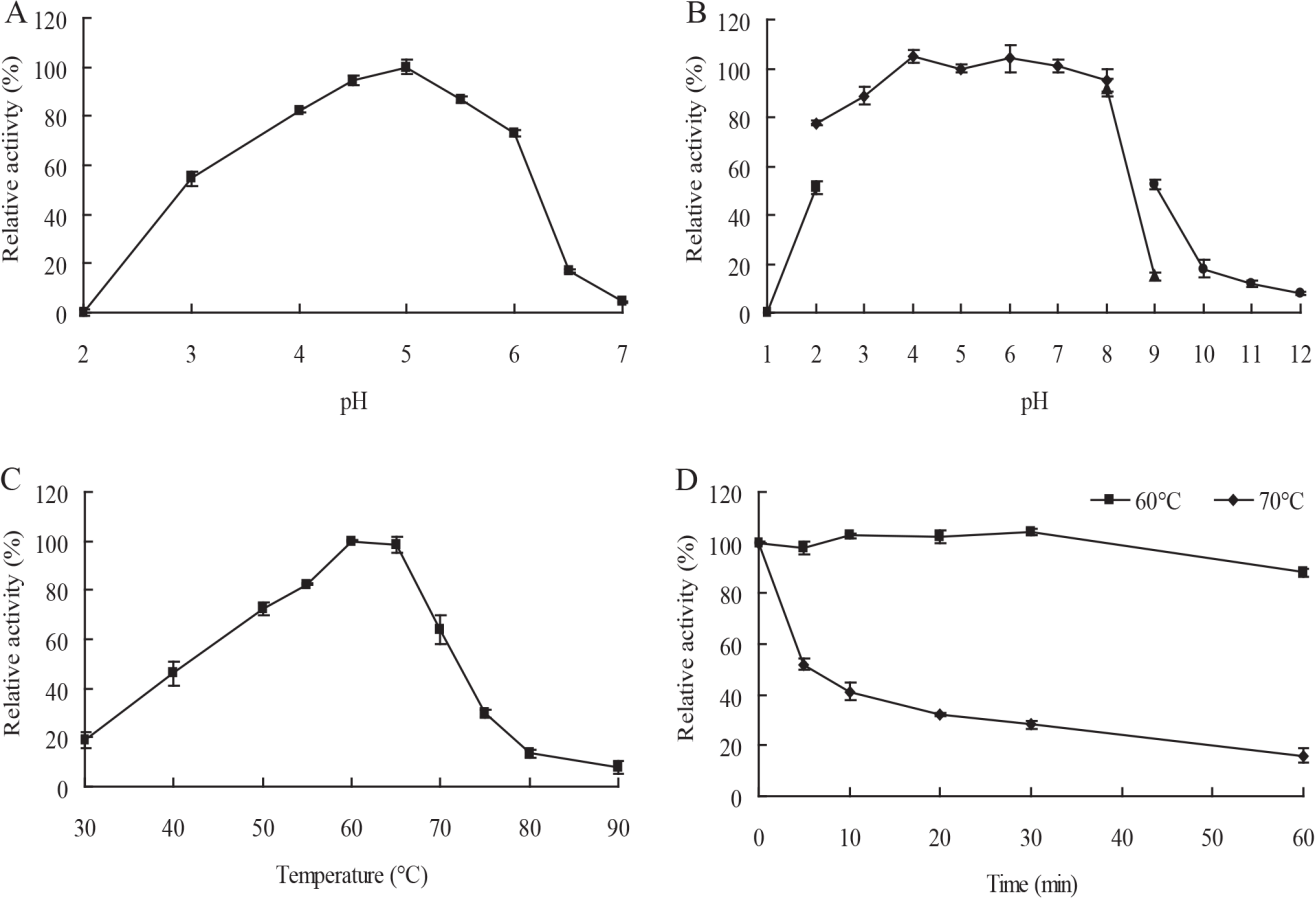
Characterization of purified recombinant Cel7A. (**A)** Effect of pH on enzyme activity assayed at 60°C. (**B)** pH stability. The enzyme activity was assayed after 1-h incubation at 37°C and different pH values. (**C)** Effect of temperature on enzyme activity at optimal pH. (**D)** Thermostability assay at 60°C and 70°C. Each value in the panel represents the means ± SD (*n* = 3).

Recombinant Cel7A had optimal activity at pH 5.0, and exhibited >50% activity between pH 3.0 and 6.0 (Fig 2A). Cel7A was stable over a broad pH range, retaining almost 100% of the initial activity after 1-h incubation at pH 3.0 to 8.0 (Fig 2B). The optimal temperature of Cel7A was 60°C when assayed at pH 5.0, and the enzyme remained >50% activity between 50°C and 70°C (Fig 2C). Cel7A was highly stable at 60°C, retaining almost 100% of the activity after 1-h incubation; After incubation at 70°C for 1 h, it retained 16.1% of the initial activity (Fig 2D).

We also tested the effects of various metal cations and chemical reagents (5 mM) on Cel7A activity (Table 2). Among those tested, FeCl_3_, CaCl_2_, CrCl_3_, NaCl, KCl, LiCl, ZnCl_2_, MgCl_2_, NiCl_2_, CoCl_2_, CuCl_2_, and β-mercaptoethanol modestly stimulated or had no effect on the Cel7A activity, whereas AgNO_3_ and the anionic surfactant SDS abolished the activity. Pb(CH_3_COO)_2_, MnCl_2_, and EDTA inhibited the enzyme activity.

**Table 2:** The effect of metal cations and reagents (5 mM) on the activity of recombinant Egl7A.

Chemicals	Relative activity (%) ^a^	Chemicals	Relative activity (%)
Control	100 ± 1	Co^2+^	90 ± 6
Fe^3+^	132 ± 6	Cu^2+^	90 ± 5
Ca^2+^	119 ± 3	Na^+^	65 ± 5
Cr^3+^	114 ± 4	Pb^2+^	56 ± 3
K^+^	113 ± 2	Mn^2+^	44 ± 6
Li^+^	111 ± 1	Ag^+^	0
Zn^2+^	108 ± 4	β-mercaptoethanol	104 ± 2
Mg^2+^	95 ± 5	EDTA	76 ± 4
Ni^2+^	94 ± 4	SDS	5 ± 1

^a^ Values represent the mean ± SD (n = 3) relative to untreated control sample

### Hydrolysis products of barley β-glucan and CMC-Na by Cel7A

**Fig 3:**
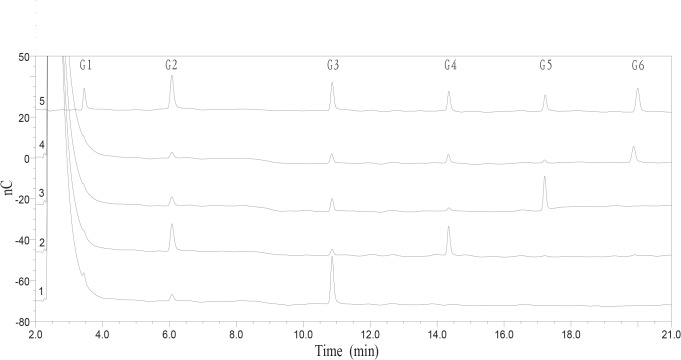
HPAEC analyses of the hydrolysis products of cellooligosaccharide. 1, cellotriose; 2, cellotetraose; 3, cellopentaose; 4, cellohexaose; 5, cellooligosaccharide standards: G1, glucose; G2, cellobiose; G3, cellotriose; G4, cellotetraose; G5, cellopentaose; G6, cellohexaose.

**Fig 4:**
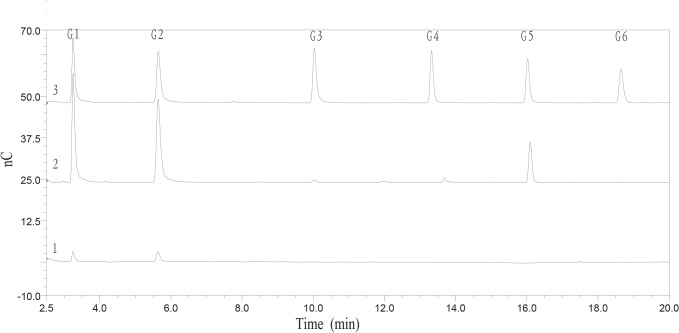
HPAEC analyses of the hydrolysis products of barley β-glucan and CMC-Na. 1, The hydrolysis products of CMC-Na; 2, The hydrolysis products of barley β-glucan; 3, the cellooligosaccharide standards: G1, glucose; G2, cellobiose; G3, cellotriose; G4, cellotetraose; G5, cellopentaose; G6, cellohexaose.

To explore the biochemical properties of Cel7A, the activity of this enzyme against cello-oligosaccharides was determined. Cel7A failed to degrade cellobiose (data not shown), but produced glucose and cellobiose from cellotriose. Moreover, shorter chain oligosaccharides were generated from longer chain cello-oligosaccharides (Fig 3). The results indicated that Cel7A is an endo-acting enzyme that can randomly cleave the β-linkages. The hydrolysis products of barley β-glucan and CMC-Na were analyzed by HPAEC-PDA after 12 h incubation with Cel7A at 37°C. The main hydrolysis products of barley β-glucan were 38.9% glucose, 41.6% cellobiose, 1.0% cellotriose, 0.2% cellotetraose, 14.5% cellopentaose, 0.2% cellohexaose, and 3.6% other oligosaccharides. The mass composition of the hydrolysis products from CMC-Na was 40.6% glucose, 50.3% cellobiose, and 9.1% other oligosaccharides (Fig 4). The time course analysis of barley β-glucan hydrolysis revealed that Cel7A displayed substantial accumulation of glucose, cellobiose, cellopentaose, and transient accumulation of cellotriose and cellohexaose (S2 Fig).

### Effects of Cel7A on mash viscosity reduction

Under simulated mashing conditions, after incubation with 200 U of purified Cel7A, the results showed Cel7A had capacity to decrease the viscosity of mash (9.1%) and increase the filtration rate of mash (24.6%), indicating that Cel7A had positive effect on the mashing performance.

## Discussion

Several fungal GH7 endo-1,4-β-glucanases including Cel7A in this work have been successfully expressed in *P*. *pastoris* for industrial purposes [10, 21−23]. The molecular weight of recombinant Cel7A found in SDS-PAGE is much higher than its calculated molecular mass. After N-deglycosylation with Endo H, the molecular weight decreased only slightly. Based on sequence analysis, Cel7A contained a typical hydrophobic signal sequence at residues 1–20, a catalytic domain of GH7 (residues 21–400), a Thr/Ser-rich linker region at residues 401–435, and a CBM 1 at residues 436–469 (S3 Fig). Of the 34 residues in the linker region, 71% residues are Thr and Ser, some of which might serve as potential sites for O-linked glycosylation (http://www.cbs.dtu.dk/services/NetOGlyc/). The O-glycosylation most likely accounts for the higher molecular weight [24]. The same observations have been found when heterologously expressed MtEG7a from *M*. *thermophila* ATCC 42464 [21], Cel7B from *P*. *decumbens* [23], StCel61a from *Sporotrichum thermophile* [25], and Man5XZ3 from *A*. *nidulans* XZ3 [26], which all have Thr/Ser rich linker regions.

To explore the mode action of Cel7A, we analyzed its activity against cellooligosaccharides. As shown in Fig 3, Cel7A released glucose, cellobiose, cellotriose, cellotetraose, and cellopentaose from cellohexaose, glucose, cellobiose, cellotriose, and cellotetraose from cellopentaose, and glucose, cellobiose, and cellotriose from cellotetraose, respectively. We conclude that Cel7A represents an endo-random acting β-1,4-glucanase. Like most GH 7 endo-1,4-β-glucanases, Cel7A has higher activity on barley β-glucan than CMC-Na. The reason might be that CMC is highly substituted with methoxy side chains that interfere with the enzyme activity [27]. In addition, the susceptibility of soluble polymers to Cel7A was determined to be in the order of barley β-glucan > lichenin > CMC-Na > laminarin > locust bean galactomannan > beechwood xylan. Cel7A can hydrolyze not only mixed β-1,4- and β-1,3-glucosidic linkages but also β-1,4 or β-1,3-glucosidic linkages, thus it belongs to the group of non-specific β-1,4-glucanase with weak β-1,3-glucanase activity. The inactivity of Cel7A on *p*NPG and *p*NPC suggests that it has no β-glucosidase and cellobiohydrolase activities. In addition, Cel7A has weak activity on insoluble polymers, including Avicel, PASC, and filter paper. There are a few reports of GH 7 endoglucanases that have very low activity on Avicel, such as MtEG7a from *M*. *thermophila* ATCC 42464 [21] and Cel7B from *P*. *decumbens* [23]. However, several glucanases including Bgl7A from *Bispora* sp. MEY-1 [22], CelG5 from *Phialophora* sp. G5 [28], and Egl7A from *T*. *emersonii* CBS394.64 [10] cannot hydrolyze Avicel.

Most fungal endo-β-1,4-glucanases of GH7 show optimal activities at acidic pH 4–6 [10, 22, 23]. However, MtEG7a from *M*. *thermophila* ATCC 42464 [21] and some bacterial endo-β-1,4-glucanases have activity at pH >8.0. Cel7A exhibits similar properties to fungal counterparts, having maximal activity at pH 5.0 and exhibiting >50% of the maximum activity at pH 3.0–6.0. Moreover, Cel7A is highly stable over a broad pH range from acid and neutral (pH 3.0–8.0). Furthermore, Cel7A has a specific activity of 2,020 U mg^–1^ towards barley β-glucan, which is significantly higher than Cel7B from *P*. *decumbens* (582 U mg^–1^) [23] and MtEG7a from *M*. *thermophila* ATCC 42464 (298 U mg^–1^) [21], but lower than Bgl7A from *Bispora* sp. MEY-1 (4,040 U mg^–1^) [22] and Egl7A from *T*. *emersonii* CBS394.64 (11,299 U mg^–1^) [10]. Considering its optimal activity at acid and mesophilic conditions, and relatively high expression level and specific activity, Cel7A meets the requirement of brewing industry for candidate additives, and its effect on the mash filtration rate and viscosity was assessed then. In comparison to other endo-β-1,4-glucanases (S2 Table) [29], Cel7A even at a lower enzyme concentration can reduce the mash viscosity more significantly than the β-glucanase from *R*. *microsporus* var. *microsporus* and two commercial enzymes A and B [19], but lower than Egl7A from *T*. *emersonii* CBS394.64 [10].

## Conclusion

A GH7 β-1,4-glucanase was identified in *N*. *fischeri* P1 and produced in *P*. *pastoris* at high levels. Cel7A has favorable properties including high activity and good stability at acidic pH, high catalytic efficiency towards barley β-glucan, good thermostability, broad substrate specificity, and is effective at reducing mash viscosity. This enzyme represents a good candidate in the basic research and various industrial applications, especially in the brewing industry.

## Supporting Information

S1 Fig Multiple alignment of the Cel7A protein sequence and homologous enzymes. (DOCX)

S2 Fig Time course of hydrolysis of barley β-glucan by Cel7A. (DOCX)

S3 Fig The nucleotide and amino acid sequence analysis of Cel7A. (DOCX)

S1 Table Summary of the purification of recombinant Cel7A. (DOCX)

S2 Table Effect of endo-β-1,4-glucanases on the viscosity and filtration rate of mash. (DOCX)
