# Supplementary material for: A Novel GH7 Endo-β-1,4-Glucanase from Neosartorya fischeri P1 with Good Thermostability, Broad Substrate Specificity and Potential Application in the Brewing Industry
Source: PLoS One. 2015 Sep 11;10(9):e0137485. doi: 10.1371/journal.pone.0137485 (PMC4567307; doi:10.1371/journal.pone.0137485)
Supplement: S1 Fig — (DOCX) [file pone.0137485.s001.docx]

*P. decumbens* 1 HWIHTANGYDSCTTSSG-VDPTLCPDATTCSKNCVIEPANYTSSGVATSGDSLTLHQYVK
*N. fischeri* 1 HWIHTVNGYTSCTTSSG-VDSTLCPDEATCAKNCVIEPANYTSAGVATSGDSLTMYQYVQ
*T. reesei* 1 RWMHDAN-YNSCTVNGG-VNTTLCPDEATCGKNCFIEGVDYAASGVTTSGSSLTMNQYMP
*Bispora* sp. 1 HPIDDIHTNESCETSSGGVNTTICPTVEVCAENCALEGVNYASHGVYTNGDSVTLRQYLN

*P. decumbens* 60 SD-GTYNNASPRVYLLGPDG-DYVLMKLLGQELTFDVDLSTLPCGENGALYLSEMSGSGG
*N. fischeri* 60 NN-GVYTNASPRLYLLGPDK-DYVMLKLLGQELSFDVDLSTLPCGENGALYLSEMSASGG
*T. reesei* 59 SSSGGYSSVSPRLYLLDSDG-EYVMLKLNGQELSFDVDLSALPCGENGSLYLSQMDENGG
*Bispora* sp. 61 ID-GTEEEVSPRVYLLDPSGRDYEILKLLNQEISFTVDVSNLPCGMNGALYLTSMDASGG
 *** ***
*P. decumbens* 118 RNANNKGGAAYGSGYCDAQCPLETWKNGTLVPGGQAYCCNEMDILEGNSAANSYTPHPCS
*N. fischeri* 118 RNEYNTGGAEYGSGYCDAQCPVIAWKNGTLNTSGASYCCNEMDILEANSRANSYTPHPCS
*T. reesei* 118 ANQYNTAGANYGGGYCDAQCPVQTWRNGTLNTSHQGFCCNEMDILEGNSRANALTPHSCT
*Bispora* sp. 120 RSQLNPAGATYGTGYCDAQCNAPAWINGVANLKGLGACCSEMDLWEANSEATQLTPHACN

*P. decumbens* 178 SD--------------DCDKGGCGFNPYAQGKTNYWAP--GGTVDTSKPFTINTQFITND
*N. fischeri* 178 AT--------------DCDKGGCGFNPYALGQKSYWGP--GGTVDTSKPFTITTQFITND
*T. reesei* 178 AT--------------ACDSAGCGFNPYGSGYKSYYGP--GDTVDTSKTFTIITQFNTDN
*Bispora* sp. 180 VTGFYECSGAACGSNGVCDKDGCGFNPYGLGDHSFYGPSVTDTIDTKKPFTVVTQFLTSD

*P. decumbens* 222 GTTTGTLTEIRRQYIQNGKVIANAKSSAG---VDSIKEAWCESVDGAAATFGGLTTMGKA
*N. fischeri* 222 GTTTGTLSEIRRQYIQNGKVIANAVSSAG---VSSITEDWCTSVDSSAAIFGGLTTMGKA
*T. reesei* 222 GSPSGNLVSITRKYQQNGVDIP--STQPG---GDTISS--CP----SASAYGGLATMGKA
*Bispora* sp. 240 GTSTGTLSQIRRLYLQNGKVIQNAKVDFDNSTLDSITPAYCEATAATFEAEGGFPQMGKA

*P. decumbens* 279 LGRGMVLIFSIWNDVSGNMNWLDSGSNGPCSSTEGSPTNLIAQNPDTHVVFSNIRWGDIG
*N. fischeri* 279 LGRGMVLIFSIWNDASGFMNWLDSGNSGPCSSTEGNPDLIKAQNPTTHVVFSNIRWGDIG
*T. reesei* 271 LSSGMVLVFSIWNDNSQYMNWLDSGNAGPCSSTEGNPSNILANNPNTHVVFSNIRWGDIG
*Bispora* sp. 300 LEMGMVLIFSIWNDPSAFMNWLDSGTAGPCNSTQGNPALIEAENPGTSVTFSNIKWGDIG

*P. decumbens* 339 STFNAPGGSGSSSSTTTTTTVATTKATSTTTKISTTTTAGGATQTQWGQCGGQGYTGPTA
*N. fischeri* 339 STFKGSDDSG----TTTTKTTTTKTSTSTTSTKTTTSTAPGATQTHYGQCGGQGWTGPTA
*T. reesei* 331 STTNS---------TAPPPPPASSTTFSTTRRSSTTSSSPSCTQTHWGQCGGIGYSGCKT
*Bispora* sp. 360 TTYSG--------------------------------------------SGSGGWN----

*P. decumbens* 399 CVSGTTCKAQNPYYSQCL
*N. fischeri* 395 CASPYTCKAQNQWYSQCL
*T. reesei* 382 CTSGTTCQYSNDYYSQCL
*Bispora* sp. ------------------

**S1 Fig. Multiple sequence alignment of the Cel7A and homologous enzymes** **using the ClustalW program.** The sequences include deduced Cel7A from *N. fischeri* P1, the endo-β-glucanases Bgl7A from *Bispora* sp. MEY-1 (FJ695140), endoglucanase I from *Trichoderma reesei* (P07981), Cel7B from *Penicillium decumbens* (EU339127) and Cel7A from *Talaromyces emersonii* CBS394.64. Identical and similar amino acids are indicated by black and gray shades, respectively. The catalytic glutamate residues are indicated by asterisks
