# Supplementary material for: A Novel GH7 Endo-β-1,4-Glucanase from Neosartorya fischeri P1 with Good Thermostability, Broad Substrate Specificity and Potential Application in the Brewing Industry
Source: PLoS One. 2015 Sep 11;10(9):e0137485. doi: 10.1371/journal.pone.0137485 (PMC4567307; doi:10.1371/journal.pone.0137485)
Supplement: S2 Fig — (DOCX) [file pone.0137485.s002.docx]

**S2 Fig.** **Time course of hydrolysis of barley β-glucan by Cel7A.** 1, the barley β-glucan incubated without enzyme for 12 h; 2–7, the hydrolysate with enzyme treatment over 20 min; 8, the cellooligosaccharide standards: G1, glucose; G2, cellobiose; G3, cellotriose; G4, cellotetraose; G5, cellopentaose; G6, cellohexaose
