# Supplementary material for: A Novel GH7 Endo-β-1,4-Glucanase from Neosartorya fischeri P1 with Good Thermostability, Broad Substrate Specificity and Potential Application in the Brewing Industry
Source: PLoS One. 2015 Sep 11;10(9):e0137485. doi: 10.1371/journal.pone.0137485 (PMC4567307; doi:10.1371/journal.pone.0137485)
Supplement: S3 Fig — (DOCX) [file pone.0137485.s003.docx]

1. ATGGACTCCAAAAGAGGCATTGTGGCCGCTGTGCTGGCCTTGCTTTCTGTCGTTTCTGCGCAACAACCCGCCACAAGTTCTGCTGGTAAC

M D S K R G I V A A V L A L L S V V S A Q Q P A T S S A G N

[ Signal sequence ]

91 CCCAAGCTTACAACATACAAATGTACCACTGCCGGCGGCTGTGTTGCGCAAGATACATCTGTTGTTCTTGATTGGGGCTCCCACTGGATC

P K L T T Y K C T T A G G C V A Q D T S V V L D W G S H W I

181 CACACGGTCAATGGGTATACATCATGCACCACATCGTCCGGAGTCGACAGTACCCTGTGTCCTGATGAGGCCACCTGCGCGAAGAACTGT

H T V N G Y T S C T T S S G V D S T L C P D E A T C A K N C

271 GTGATCGAGCCGGCCAACTACACCAGCGCCGGTGTGGCCACCTCGGGTGACTCTCTGACTATGTACCAATATGTTCAGAACAACGGCGTC

V I E P A N Y T S A G V A T S G D S L T M Y Q Y V Q N N G V

361 TACACCAACGCCTCGCCTCGCCTCTACCTCCTCGGCCCAGACAAGGACTATGTCATGCTGAAGCTGCTAGGCCAGGAGCTCTCCTTCGAC

Y T N A S P R L Y L L G P D K D Y V M L K L L G Q E L S F D

451 GTGGACCTGTCCACACTCCCCTGCGGCGAAAACGGTGCCCTGTATCTCTCCGAAATGAGCGCCAGCGGTGGTCGCAACGAATACAACACC

V D L S T L P C G E N G A L Y L S E M S A S G G R N E Y N T

541 GGAGGCGCCGAGTACGGCTCTGGCTACTGTGACGCGCAATGCCCCGTGATTGCCTGGAAGAACGGCACCCTCAACACGAGCGGCGCAAGC

G G A E Y G S G Y C D A Q C P V I A W K N G T L N T S G A S

631 TACTGCTGCAACGAGATGGACATCCTCGAGGCCAACTCCCGCGCCAACTCGTACACCCCGCACCCCTGCAGCGCAACGGACTGTGACAAG

Y C C N E M D I L E A N S R A N S Y T P H P C S A T D C D K

721 GGCGGATGCGGCTTCAACCCATACGCTCTAGGCCAGAAGAGCTACTGGGGGCCCGGCGGCACCGTCGACACTTCTAAGCCCTTCACCATC

G G C G F N P Y A L G Q K S Y W G P G G T V D T S K P F T I

811 ACCACGCAGTTCATCACCAACGACGGCACCACCACCGGCACTTTGTCCGAAATCCGCAGACAGTACATCCAGAACGGCAAGGTGATCGCC

T T Q F I T N D G T T T G T L S E I R R Q Y I Q N G K V I A

901 AACGCCGTTTCCTCCGCTGGCGTCAGCTCCATTACCGAGGACTGGTGCACGTCCGTCGACAGCTCGGCCGCCATCTTTGGCGGACTGACC

N A V S S A G V S S I T E D W C T S V D S S A A I F G G L T

991 ACCATGGGCAAGGCGCTGGGCCGCGGGATGGTCCTCATCTTCAGCATCTGGAACGATGCCAGCGGCTTCATGAACTGGCTCGACAGCGGC

T M G K A L G R G M V L I F S I W N D A S G F M N W L D S G

1081 AATTCAGGCCCTTGCAGCAGCACCGAGGGGAACCCGGACCTGATCAAGGCGCAGAATCCCACGACCCACGTGGTCTTCTCTAATATCCGC

N S G P C S S T E G N P D L I K A Q N P T T H V V F S N I R

1171 TGGGGAGATATCGGATCGACTTTCAAGGGTTCTGATGACTCGGGTACGACGACCACTAAGACCACAACTACCAAGACGTCCACGTCCACC

W G D I G S T F K G S D D S G T T T T K T T T T K T S T S T

[ Linker region

1261 ACGTCAACCAAGACTACCACTAGCACTGCGCCCGGGGCGACGCAAACTCACTATGGACAGTGCGGTGGACAGGGCTGGACTGGACCCACG

T S T K T T T S T A P G A T Q T H Y G Q C G G Q G W T G P T

] [ CBM 1

1351 GCTTGTGCATCGCCCTACACTTGCAAGGCTCAGAATCAGTGGTACTCACAGTGTCTGTAG

A C A S P Y T C K A Q N Q W Y S Q C L *

]

**S3 Fig.** **The nucleotide and amino acid sequence analysis of Cel7A.** The signal sequence is indicated by a double solid line, the linker region is indicated by a dotted line, and the CBM1 motif is indicated by a solid line.
