# Supplementary material for: A Novel GH7 Endo-β-1,4-Glucanase from Neosartorya fischeri P1 with Good Thermostability, Broad Substrate Specificity and Potential Application in the Brewing Industry
Source: PLoS One. 2015 Sep 11;10(9):e0137485. doi: 10.1371/journal.pone.0137485 (PMC4567307; doi:10.1371/journal.pone.0137485)
Supplement: S1 Table — (DOCX) [file pone.0137485.s004.docx]

**S1 Table.** **Summary of the purification of recombinant Cel7A.**

| Steps | Total activity | Total protein | Specific activity | Yield | Purification  (fold) |
| --- | --- | --- | --- | --- | --- |
|  | (U) | (mg) | (U mg^−1^) | (%) |  |
| Crude extract | 5351 | 48 | 111 | 100.0 | 1.0  4.1  18.2 |
| Ultrafiltration | 4897 | 11 | 455 | 91.5 |  |
| Sephadex G-25 | 2020 | 1 | 2020 | 37.7 |  |
