# Supplementary material for: A Novel GH7 Endo-β-1,4-Glucanase from Neosartorya fischeri P1 with Good Thermostability, Broad Substrate Specificity and Potential Application in the Brewing Industry
Source: PLoS One. 2015 Sep 11;10(9):e0137485. doi: 10.1371/journal.pone.0137485 (PMC4567307; doi:10.1371/journal.pone.0137485)
Supplement: S2 Table — (DOCX) [file pone.0137485.s005.docx]

**S2 Table.** **Effect of endo-β-1,4-glucanases on the viscosity and filtration rate of mash.**

| Enzymes | Filtration time reduction (%) | Viscosity reduction (%) | Reaction conditions | References |
| --- | --- | --- | --- | --- |
| Cel7A (99 µg) | 24.6 | 9.1 | 45°C for 30 min, 50°C for 30 min, 60°C for 60 min, 70°C for 15 min | This study |
| CelG5 (54 µg) | 17.7 | 5.3 | 45°C for 30 min, 50°C for 30 min, 60°C for 60 min, 70°C for 30 min | 28 |
| Egl7A (4.4 µg) | －^a^ | 12.4 | 50°C for 30 min,  60°C for 30 min,  70°C for 60 min,  80°C for 30 min | 10 |
| Agl9A (215 µg) | 26.7 | 6.1 | 45°C for 30 min,  50°C for 30 min,  60°C for 60 min,  70°C for 15 min | 29 |
| 1,3-1,4-β-Glucanase (110 µg) | 20.4 | 4.7 | 45°C for 30 min,  50°C for 10 min,  60°C for 15 min,  70°C for 60 min | 19 |
| Commercial enzyme A (394 µg) | 29.9 | 6.3 | 45°C for 30 min,  50°C for 10 min,  60°C for 15 min,  70°C for 60 min | 19 |
| Commercial enzyme B (373 µg) | 35.8 | 6.4 | 45°C for 30 min,  50°C for 10 min,  60°C for 15 min,  70°C for 60 min | 19 |

^a^－, not detected
